# Policy Spotlight Effects on Critical Time-Sensitive Diseases: Nationwide Retrospective Cohort Study on Taiwan’s Hospital Emergency Capability Categorization Policy

**DOI:** 10.2196/54651

**Published:** 2025-03-25

**Authors:** Chih-Yuan Lin, Chih-Ching Liu, Yu-Tung Huang, Yue-Chune Lee

**Affiliations:** 1 Department of Neurology Taipei City Hospital Linsen Chinese Medicine Branch Taipei City Taiwan; 2 Institute of Health and Welfare, College of Medicine, Yangming Campus National Yang Ming Chiao Tung University, Taipei, Taiwan Taipei City Taiwan; 3 Department of Health Care Management National Taipei University of Nursing and Health Science Taipei Taiwan; 4 National Center for Geriatrics and Welfare Research National Health Research Institutes Yunlin Taiwan

**Keywords:** categorization of hospital emergency capability, quality, time-sensitive diseases, emergency care, difference-in-differences

## Abstract

**Background:**

Taiwan’s categorization of hospital emergency capability (CHEC) policy is designed to regionalize and dispatch critical patients. The policy was designed in 2009 to improve the quality of emergency care for critical time-sensitive diseases (CTSDs). The CHEC policy primarily uses time-based quality surveillance indicators.

**Objective:**

We aimed to investigate the impact of Taiwan’s CHEC policy on CTSDs.

**Methods:**

Using Taiwan’s 2005 Longitudinal Health Insurance Database, this nationwide retrospective cohort study examined the CHEC policy’s impact from 2005 to 2011. Propensity score matching and difference-in-differences analysis within a generalized estimating equation framework were used to compare pre- and postimplementation periods. The study focused on acute ischemic stroke (AIS), ST-segment elevation myocardial infarction (STEMI), septic shock, and major trauma. AIS and STEMI cases, monitored with time-based indicators, were evaluated for adherence to diagnostic and treatment guidelines as process quality measures. Mortality and medical use served as outcome indicators. Major trauma, with evolving guidelines and no time-based monitoring, acted as a control to test for policy spotlight effects.

**Results:**

In our cohort of 9923 patients, refined through 1:1 propensity score matching, 5566 (56.09%) were male and were mostly older adults. Our analysis revealed that the CHEC policy effectively improved system efficiency and patient outcomes, resulting in significant reductions in medical orders (–7.29 items, 95% CI –10.09 to –4.48; *P*<.001), short-term mortality rates (–0.09%, 95% CI –0.17% to –0.02%; *P*=.01) and long-term mortality rates (–0.09%, 95% CI –0.15% to –0.04%; *P*=.001), and total medical expenses (–5328.35 points per case, 95% CI –10,387.10 to –269.60; *P*=.04), despite a modest increase in diagnostic fees (376.37 points, 95% CI 92.42-660.33; *P*=.01). The CHEC policy led to notable increases in diagnostic fees, major treatments, and medical orders for AIS and STEMI cases. For AIS cases, significant increases were observed in major treatments (β=0.77; 95% CI 0.21-1.33; *P=*.007) and medical orders (β=15.20; 95% CI 5.28-25.11; *P*=.003) compared to major trauma. In STEMI cases, diagnostic fees significantly increased (β=1983.75; 95% CI 84.28-3883.21; *P*=.04), while upward transfer rates significantly decreased (β=–0.59; 95% CI –1.18 to –0.001; *P*=.049). There were also trends toward increased major treatments (β=0.30; 95% CI –0.03 to 0.62, *P*=.07), medical orders (β=11.92; 95% CI –0.90 to 24.73; *P*=.07), and medical expenses (β=24,275.54; 95% CI –640.71 to 4,991,991.78; *P*=.06), although these were not statistically significant. In contrast, no significant changes were identified in process or outcome quality indicators for septic shock. These findings suggest policy spotlight effects, reflecting a greater emphasis on diseases directly prioritized under the CHEC policy.

**Conclusions:**

The CHEC policy demonstrated the dual benefits of reducing costs and improving patient outcomes. We observed unintended consequences of policy spotlight effects, which led to a disproportionate improvement in guideline adherence and process quality for CTSDs with time-based surveillance indicators.

## Introduction

### Background

The Agency for Healthcare Research and Quality proposed the concept of time-sensitive diseases [1] using scientific data to maintain up-to-date guidelines and launched the Get with the Guidelines campaign to establish it as the basis for surveillance indicators of process and outcome quality [2]. Critical time-sensitive diseases (CTSDs) refer to life-threatening illnesses or injuries that require immediate emergency care, where rapid intervention is paramount to mitigate morbidity and mortality [3]. The various guidelines for managing time-sensitive events emphasize the crucial importance of time. In the context of acute ischemic stroke (AIS), the “time is brain” [4] goal focuses on the timely reperfusion treatments, including intravenous thrombolysis and mechanical thrombectomy; in ST-segment elevation myocardial infarction (STEMI), the “time is muscle” goal focuses on early reperfusion [5]; in septic shock events, the “early goal” focuses on early resuscitation [6]; and in major trauma cases, the “golden hour” goal focuses on the window of opportunity in which patients can undergo rescue operations [7].

The American Medical Association issued the categorization of hospital emergency capability (CHEC) guidelines [8] to classify hospitals according to their emergency care capabilities, thereby regionalizing and providing emergency medical services with references for transport emergency patients to the nearest appropriate hospitals [9], aiming to reduce preventable deaths. Most studies investigating the effects of this categorization, designation, and regionalization policy reported positive findings [10,11]. However, these studies mainly focused on a single disease entity [10,11] or region [11]. The CHEC policy often implements rigid time-based surveillance indicators. These indicators can affect disease-specific guideline adherence in clinical practice because they may reshape the behaviors of emergency department (ED) medical providers [12]. This phenomenon is related to the so-called policy spotlight effects, which influence medical care providers’ assessment of how others perceive them [13]. More specifically, the policy spotlight effects refer to the perception of medical care providers regarding how policy makers interpret surveillance indicators and adjust their process-related behaviors accordingly [14]. Current emergency care policies often use time-based criteria as process quality indicators, which may exacerbate the policy spotlight effects [13]; however, the unintended effects or safety concerns generated by these effects remain unclear. Therefore, our study targeted 4 CTSDs: AIS, STEMI, septic shock, and major trauma [7]. Our research hypothesizes that emergency care providers might inadvertently give more attention to diseases under active surveillance while potentially neglecting those not thoroughly incorporated in this observation. This focus might be based on their perception of observer expectations [15].

### Objective

We aimed to examine the effects of the CHEC policy on process quality and outcomes for CTSDs, addressing three research questions: (1) How does the CHEC policy impact the quality of diagnosis, treatment, and outcomes for these diseases? (2) How do policy spotlight effects influence the prioritization of diseases under active surveillance and impact emergency care providers’ behaviors in this context? and (3) What are the potential consequences of policy spotlight effects?

## Methods

### Setting, Study Design, and Data Source

Taiwan’s National Health Insurance is a single-payer, compulsory social insurance system that primarily operates on a fee-for-service basis. This study is based on the National Health Insurance 2005 Longitudinal Health Insurance Database (LHID2005), which contains 1 million random cases, including medical records and hospital information, collected since 1995. The LHID2005 was validated to represent medical use, diagnosis and treatment process, and outcome quality for CTSDs [16].

This nationwide retrospective cohort study uses propensity score matching (PSM) and difference-in-differences (DID) analysis to evaluate the impact of the CHEC policy on CTSD care quality and outcomes. The CHEC policy was initiated in August 2009, which integrated 190 hospitals into a network focusing on acute conditions such as stroke, myocardial infarction, major trauma, and perinatal care [17]. We divided our analysis into 2 periods: before CHEC (August 1, 2005, to July 31, 2009) and after CHEC (August 1, 2009, to July 31, 2011). This division aimed to assess the CHEC policy’s effects distinctly from the ED quality improvement plan introduced in 2012. Well-established guidelines exist for AIS, STEMI, and septic shock. In contrast, the guidelines for major trauma are continuously evolving due to the variability in injury mechanisms, locations, and severity. Moreover, AIS and STEMI events are stringently monitored under the CHEC policy with specific time-based quality indicators, whereas septic shock and major trauma events are not (Table 1).

**Table 1:** Critical time-sensitive diseases and categorization hospital emergency capability (CHEC) policy indicators in Taiwan [18].

Quality indicator	Acute ischemic stroke	ST-segment elevation MI^a^	Septic shock	Major trauma
Guidelines development	Well developed	Well developed	Well developed	Developing
Major diagnosis indicator	Brain imaging (eg, CT^b^ and MRI^c^)	EKG^d^	Blood culture	Image study
Major treatment indicator	Intravenous TPA^e^	PCI^f^	Antibiotics	Rescue operation
Guideline’s major goal	Early thrombolysis Time is brain	Early reperfusion Time is muscle	Goal-directed therapy Early goal	Rescue operation (Golden hour)
Guideline’s time-based criteria	60 min	90 min	3 to 6 h	1 h
CHEC policy indicators	Stroke team NIHSS^g^ score evaluation Intravenous TPA	PCI team Give aspirin and clopidogrel	ICUS^h^ critical care team	Trauma team ISSi evaluation
CHEC policy time-based criteria	Neurologist consultation in <30 min Door to CT in <30 min Door to CT read in <45 min Onset to needle in <3 h	Cardiologist consultation in <30 min Door to EKG in <10 min Door to needle in <30 min Door to balloon in <90 min	Admission in <24 h	Trauma team activation in <30 min

^a^MI: myocardial infarction.

^b^CT: computed tomography.

^c^MRI: magnetic resonance imaging.

^d^EKG: electrocardiography.

^e^TPA: tissue plasminogen activator.

^f^PCI: percutaneous coronary intervention.

**^g^**NIHSS: National Institute of Health Stroke Scale or Score.

^h^ICU: intensive care unit.

^i^ISS: Injury Severity Score.

Thus, we selected major trauma events as a reference for our study because they were not monitored under the CHEC policy with rigid indicators. We adopted pre- and postimplementation of a CHEC policy, using 1:1 PSM to control for confounding variables. We used the DID estimation approach to estimate the association of the CHEC policy on process and outcomes for AIS and STEMI. For the counterfactual, we used major trauma cases unexposed to the clinical guideline or CHEC policy time-based quality indicators as the basis for comparison.

### Identification of Study Cohort

This study identified CTSDs based on ED visits accompanied by a primary diagnosis using the appropriate disease code. The identification of AIS (ie, codes 433 and 434), STEMI (ie, code 410), and septic shock (ie, codes 038, 785, and 995) was based on the *International Classification of Diseases, Ninth Revision, Clinical Modification* (*ICD‐9‐CM*). Major trauma cases were classified following the American Academy of Surgery Committee guidelines (ie, codes 800-959) [19]. Due to the absence of trauma severity data in the LHID2005 database, primary *ICD-9-CM* codes served as our initial method for identifying major trauma incidents. This identification was further refined by including cases where patients received rescue surgery or were admitted to the intensive care unit, serving as additional criteria for major trauma [20]. We excluded cases before the study period and those without hospital or patient sociodemographic information. We also excluded hospitals with a volume of <5 CTSD cases per year [21]. We used the date of the first ED visit as the index date.

### Definition of Variables

The independent variable in this study was exposure to the CHEC policy intervention. Events related to AIS and STEMI were subject to rigid time-based quality indicators and regular surveillance under the CHEC policy. In contrast, despite having well-developed clinical guidelines, septic shock was not included under the CHEC policy’s stringent time-based quality indicators. Similarly, major trauma cases, which lack well-developed clinical guidelines, were not subject to these policy indicators and were used as counterfactuals in this study. The dependent variables were divided into primary and secondary outcomes. Primary outcomes included guideline adherence to diagnostic and treatment process quality indicators. For diagnostic adherence, AIS was assessed by the completion of brain imaging (eg, computed tomography or magnetic resonance imaging) within 60 minutes of hospital arrival, while STEMI was evaluated based on the completion of electrocardiography within 10 minutes. For treatment adherence, AIS required the administration of intravenous thrombolysis within 3 hours of symptom onset, and STEMI was assessed according to whether percutaneous coronary intervention was performed within 90 minutes of hospital arrival. Secondary outcomes included upward transfer rates, diagnostic fees, medical orders and expenses, and mortality rates. Upward transfer rates were defined as the proportion of patients transferred from lower-level hospitals to higher-level facilities. Diagnostic fees referred to the total costs incurred from diagnostic procedures during ED care. Medical orders and expenses represented the number and costs of medical interventions performed during ED visits. Mortality rates were measured as 30-day mortality, indicating the percentage of patients who died within 30 days from the index date (the emergency visit date), and 1-year mortality, representing deaths occurring within 1 year.

Covariates included patient-related predisposing factors, such as age, sex, and occupation. The enabling factor was the insured salary, while the Charlson Comorbidity Index, calculated using *ICD-9-CM* codes from primary diagnoses recorded in inpatient and outpatient claims data up to a year before the index date, served as a measure of health needs. Furthermore, external environmental factors, including urbanization and regional emergency resources, were considered. For hospital-level variables, the input-throughput-output model of Asplin et al [22] was used. Input was gauged using the rate of ED visits with Emergency Severity Index 1, while throughput and output efficiency were assessed via the ED’s occupancy rate. This comprehensive framework ensured a robust evaluation of the CHEC policy’s impact while accounting for potential confounding variables and contextual factors.

### Statistical Analysis

Patients’ characteristics, process quality, and outcomes were presented using descriptive statistics. Continuous data were described using mean (SD), and categorical data were presented using numbers and percentages. To enhance the robustness of the outcomes, we calculated the propensity score using a multivariable logistic regression that included all baseline covariates. The standardized mean difference was calculated to confirm the balance of potential confounders at baseline between groups before and after matching. A standardized mean difference of <0.1 was considered to represent a negligible difference [23].

We evaluated the impact of the CHEC intervention on each outcome, including overall differences, the differences within individual diseases, and between-disease differences in changes from baseline (ie, group-by-disease interaction effects), using a DID framework integrated with generalized estimating equation (GEE) models. The DID approach allowed us to compare changes in outcomes between the before and after CHEC policy periods across diseases, enabling the estimation of differential effects of the intervention while controlling for time-invariant unobserved confounders. This method is particularly suitable for evaluating policy interventions by focusing on within-group changes relative to a reference group over time. The GEE model is specified as follows:

*Y_i__j_*= *β*_0_ + *β*_1_ (CHEC policy)*_j_* + *β*_2_ disease_𝑖_ + *β*_3_ (CHEC policy × disease)*_ij_* + ε*_ij_*_,_

where *Y_ij_* represents the outcome variable (eg, diagnosis indicator, treatment indicator, mortality, or medical use) for individual *i* at time *j*. *β_0_* is the intercept, capturing the baseline level of the outcome for the reference disease (ie, major trauma) in the pre-CHEC period. CHEC policy*_j_* is a binary variable (0=before CHEC, 1=after CHEC), and its coefficient *β*_1_ captures the overall impact of the CHEC policy across all diseases. Disease*_i_* is a categorical variable representing the 4 diseases (ie, AIS, STEMI, septic shock, and major trauma), with *β*_2_ estimating disease-specific differences at baseline. The interaction term (CHEC policy × disease)*_ij_*, with coefficient *β*_3_, reflects the differential impact of the CHEC policy for each disease compared to the reference group (ie, major trauma). A positive *β*_3_ value indicates a greater change in the outcome for the respective disease relative to major trauma. All statistical analyses were performed using SAS (version 9.4; SAS Institute), and statistical significance was defined as 𝑃 value of <.05.

### Ethical Considerations

This study used secondary data from the NIH LHID2005, which are fully anonymized and deidentified to protect participant privacy. The dataset contained no personal identifiers, such as names, addresses, or social security numbers, so individual informed consent was not required. The original consent provided for primary data collection, and the institutional review board approval covered secondary analyses without requiring additional consent. All analyses were conducted in compliance with relevant regulations to safeguard confidentiality, and data access was restricted to authorized researchers under institutional guidelines. The study received ethics approval from the Taiwan National Yang-Ming University Institutional Review Board (YM107035E).

## Results

### Participants’ Characteristics

**Figure 1:**
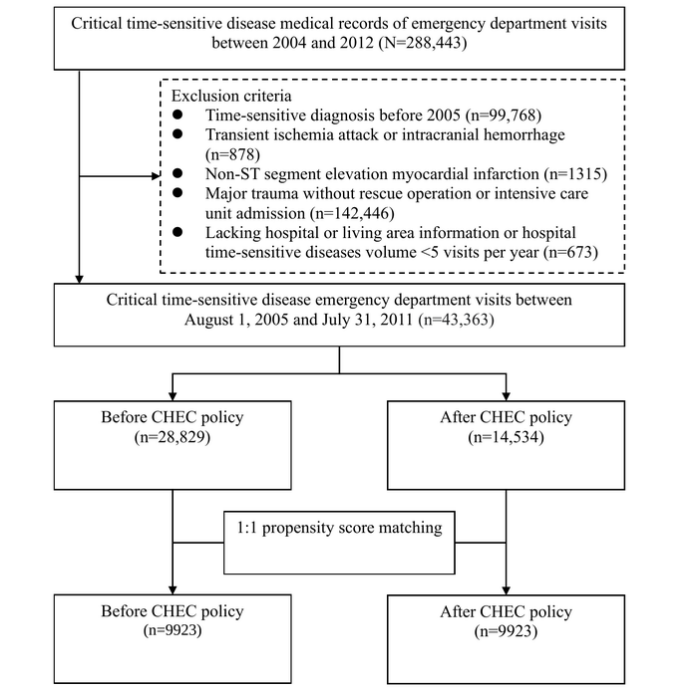
Flow diagram of the study.

During the study period, we analyzed emergency presentations related to 4 CTSDs, originally encompassing 288,443 patients. Exclusion criteria included the diagnosis of CTSDs before 2005 (n=99,768, 34.59%), patients with transient ischemic attack or intracranial hemorrhage (n=878, 0.3%), non-STEMI cases (n=1315, 0.45%), individuals with major traumas defined by *ICD-9-CM* codes that did not necessitate a rescue operation or intensive care unit admission (n=142,446, 49.38%), and cases lacking hospital or living area information or where the hospital’s volume of CTSDs was <5 visits per year (n=673, 0.23%). These criteria refined the total sample size to 43,363 (15.03%) patients. Considering the extended period before the policy intervention, this research adopted a 1:1 PSM technique, resulting in a final matched sample of 9923 patients. The flowchart and baseline table (Figure 1) display the initial count of emergency patients with CTSDs and the numbers after PSM, broken down by each of the 4 diseases. Table 2 presents the PSM of participants with CTSDs before and after the PSM. After the matching process, each variable baseline characteristic demonstrated almost complete congruity. In addition, uniformity was achieved within each disease subgroup after matching (Table S1 in Multimedia Appendix 1). In 9923 patients, the distribution for each disease before and after PSM was as follows: AIS (n=2895, 29.17%), STEMI (n=723, 7.29%), septic shock (n=5441, 54.83%), and major trauma (n=864, 8.71%).

Septic shock was the most prevalent condition, accounting for 54.83% (5441/9923) of all cases. The patient population was male-dominated (5566/9923, 56.09%), with the majority (6084/9923, 61.31%) aged ≥65 years. Nearly three-fourths of the CTSD cases (7563/9923, 76.12%) were managed in hospitals categorized as moderate or severe levels. Care provided by specialty consultants accounted for 67.9% (6738/9923) of the cases.

**Table 2:** Propensity score matched the comparison of patient characteristics before and after the categorization of hospital emergency capability (CHEC) policy. The complete table is available in Supplementary Table 2: Association of CHEC policy with process and outcome quality in four critical time-sensitive diseases (Multimedia Appendix 2).

Patient characteristics			Before propensity score matching							After propensity score matching				
			Before CHEC policy (n=28,829)		After CHEC policy (n=14,534)		ASMD^a^		Before CHEC policy (n=9923)			After CHEC policy (n=9923)		ASMD
**Sex, n (%)**														
	Female	12,345 (42.82)		6250 (43)		0.004		4357 (43.91)			4268 (43.01)		0.018	
	Male	16,484 (57.18)		8284 (57)		0.004		5566 (56.09)			5655 (56.99)		0.018	
**Age (y), n (%)**														
	≤45	4105 (14.24)		1967 (13.53)		0.021		1289 (12.99)			1306 (13.16)		0.005	
	45-64	7173 (24.88)		3795 (26.11)		0.028		2550 (25.7)			2590 (26.1)		0.009	
	≥65	17,551 (60.88)		8772 (60.36)		0.011		6084 (61.31)			6027 (60.74)		0.012	
**Charlson Comorbidity Index, n (%)**														
	≤1	13,215 (45.84)		6546 (45.04)		0.016		4473 (45.08)			4434 (44.68)		0.008	
	>1	15,614 (54.16)		7988 (54.96)		0.016		5450 (54.92)			5489 (55.32)		0.008	
**Income^b^ (NT $), n (%)**														
	≤22,800	15,961 (55.36)		6203 (42.68)		0.256		4428 (44.62)			4604 (46.4)		0.036	
	>22,800	12,868 (44.64)		8331 (57.32)		0.256		5495 (55.38)			5319 (53.6)		0.036	
**Occupation, n (%)**														
	Dependents of the insured	10,095 (35.02)		4952 (34.07)		0.020		3550 (35.78)			3326 (33.52)		0.048	
	Civil servants, teachers, and military personnel	2792 (9.68)		1529 (10.52)		0.028		986 (9.94)			1053 (10.61)		0.022	
	Nonmanual workers and professionals	2231 (7.74)		1240 (8.53)		0.029		748 (7.54)			796 (8.02)		0.018	
	Manual workers	10,250 (35.55)		5154 (35.46)		0.002		3422 (34.49)			3708 (37.37)		0.060	
	Others	3461 (12.01)		1659 (11.41)		0.019		1217 (12.26)			1040 (10.48)		0.056	
**Hospital categorization, n (%)**														
	Severe level	11,371 (39.44)		5924 (40.76)		0.027		3331 (33.57)			3502 (35.29)		0.036	
	Moderate level	10,921 (37.88)		5496 (37.81)		0.001		4030 (40.61)			4052 (40.83)		0.004	
	General level	6537 (22.68)		3114 (21.43)		0.030		2562 (25.82)			2369 (23.87)		0.045	
ESI^c^ triage level 1 and 2 rate, mean (SD)			5.8 (8.98)		16.63 (10.06)		1.136		14.01 (10.39)			14.37 (10.50)		0.034
Length of ED^d^ stay, mean (SD)			5.33 (10.11)		4.49 (9.30)		0.086		6.25 (9.30)			5.36 (10.26)		0.091
ED observation ≥1-d rate, mean (SD)			11.37 (9.97)		12.32 (10.06)		0.095		10.53 (9.34)			11.32 (10.12)		0.081
**Place of ED resources, n (%)**														
	Sufficiency	22,770 (78.98)		11,460 (78.85)		0.003		7768 (78.28)			7834 (78.95)		0.016	
	Not sufficiency	6059 (21.02)		3074 (21.15)		0.003		2155 (21.72)			2089 (21.05)		0.016	
**Time-sensitive disease,** **n (%)**														
	Acute ischemic stroke	8660 (30.04)		3814 (26.24)		0.085		2895 (29.17)			2895 (29.17)		0.000	
	ST-segment elevation MI^e^	2481 (8.61)		1141 (7.85)		0.028		723 (7.29)			723 (7.29)		0.000	
	Septic shock	14,896 (51.67)		8275 (56.94)		0.106		5441 (54.83)			5441 (54.83)		0.000	
	Major trauma	2792 (9.68)		1304 (8.97)		0.024		864 (8.71)			864 (8.71)		0.000	
**Delivery of care,** **n (%)**														
	Specialty consultant^f^	18,692 (64.84)		9245 (63.61)		0.026		6738 (67.9)			6570 (66.21)		0.036	
	Emergency physician	7788 (27.01)		4236 (29.15)		0.048		2449 (24.68)			2585 (26.05)		0.031	
	Others	2349 (8.15)		1053 (7.25)		0.034		736 (7.42)			768 (7.74)		0.012	

^a^ASMD: absolute standardized mean difference.

^b^INT $1=US $0.03057 as of February 16, 2025.

^c^ESI: Emergency Severity Index.

^d^ED: emergency department.

^e^MI: myocardial infarction.

^f^Specialty consultant: (1) acute ischemic stroke is treated by neurologists, (2) acute MI is treated by cardiologists, (3) septic shock is managed by internal medicine physicians or critical care intensivists, and (4) major trauma conditions are managed by surgeons or critical care intensivists.

### Impact of CHEC Policy Overall and on the Processes and Outcomes of the 4 Individual CTSDs Before and After Implementation

In examining individual diseases, primary diagnostic indicators for AIS, septic shock, and major trauma decreased after intervention, while only those for STEMI increased (Table 3).

Diagnostic fees increased for AIS, STEMI, and major trauma cases but decreased for septic shock cases. A similar trend was observed in the primary treatment indicators, which increased for AIS and STEMI cases and decreased for septic shock and major trauma cases. In contrast, medical orders showed a universal decline. Upward transfer rates increased for AIS and major trauma cases but decreased for STEMI and septic shock cases. Regarding outcome indicators, short-term and long-term mortality rates displayed a universal decline, except for AIS cases, which showed an increase. The medical expenses were higher for AIS and STEMI cases but lower for septic shock and major trauma cases.

In assessing the overall policy effects on 4 CTSD cohorts, the primary diagnosis indicator significantly decreased by 0.21% points (95% CI –0.29% to –0.13%; *P<*.001) and medical orders per case dropped by an average of 7.29 items (95% CI –10.09 to –4.48; *P<*.001). In comparison, diagnostic fees demonstrated an average increase of 376.37 points (95% CI 92.42-660.33; *P=*.01). The 30-day mortality rate saw a notable reduction of 0.09% points (95% CI –0.17% to –0.02%; *P=*.01), 1-year mortality significantly decreased by 0.09% points (95% CI –0.15% to –0.04%; *P=*.001), and medical expense per case significantly decreased by 5328.35 points (95% CI –10,387.10 to –269.60; *P=*.04).

**Table 3:** Comparative analysis of the individual and overall impact of categorization of hospital emergency capability (CHEC) policy effects on 4 critical time-sensitive diseases (N=9923).

Outcome			Change between before and after CHEC policy									Before CHEC policy			After CHEC policy			Multivariable model, *β*_1_^a^ (95% CI)			*P* value
			Acute ischemic stroke^b^		STEMI^c^		Septic shock^d^		Major trauma^e^												
**Process quality**																					
	Major diagnosis indicator, n (%)	–3.52		0.55		–2.28		–3.01		8682 (87.49)			8434 (84.99)			–0.21 (–0.29 to –0.13)			<.001		
	Diagnostic fees^f^	460.66		2746.6		–44.8		762.85		7166.94 (10,018.69)			7543.31 (10,833.26)			376.37 (92.42 to 660.33)			.01		
	Major treatment indicator, n (%)	0.62		2.07		–1.25		–3.12		4334 (43.68)			4272 (43.05)			–0.03 (–0.07 to 0.02)			.24		
	Medical orders per case	–0.93		–4.21		–9.67		–16.13		102.13 (109.19)			94.84 (101.07)			–7.29 (–10.09 to –4.48)			<.001		
	Upward transfer rate, n (%)	0.59		–1.24		–0.1		2.43		148 (1.49)			172 (1.73)			0.15 (–0.06 to 0.37)			.16		
**Outcomes**																					
	30-d mortality, n (%)	–0.04		–1.52		–1.84		–1.27		1631 (16.44)			1508 (15.2)			–0.09 (–0.17 to –0.02)			.01		
	1-y mortality, n (%)	0.28		–0.83		–3.64		–0.58		3242 (32.67)			3041 (30.65)			–0.09 (–0.15 to –0.04)			.001		
	Total medical expense per case	3616.16		11,219.01		−11,059.09		−13,056.54		100,875.96 (192,912.94)			95,547.60 (176,487.79)			–5328.35 (–10,387.10 to –269.60)			.04		

^a^Overall impact of the CHEC policy across all diseases.

^b^Acute ischemic stroke major diagnosis indicator: head image and major treatment indicator: intravenous tissue plasminogen activator thrombolysis.

^c^STEMI: ST-elevation myocardial infarction major diagnosis indicator: electrocardiography and major treatment indicator: percutaneous coronary intervention.

^d^Septic shock major diagnosis indicator: culture and major treatment indicator: antipathogen medication.

^e^Major trauma major diagnosis indicator: computed tomography, magnetic resonance imaging, or sonography study and major treatment indicator: rescue operation.

^f^Diagnostic fees: since Taiwan’s National Health Insurance system operates on a global budget with reimbursement based on a point system, the actual monetary value of each point fluctuates. Currently, 1 National Health Insurance point is reimbursed at <NT $0.90 (US $0.0275) per point based on the latest exchange rate (1 NT $=US $0.03057 as of February 16, 2025).

### Association of CHEC Policy With Processes and Outcome Quality in the 4 CTSDs

As presented in Table 4, model 1 examined the changes in indicators for individual diseases before and after the implementation of the CHEC policy, and the results show significant improvements in process quality measures. For AIS cases, following the implementation of the CHEC policy, there was a significant decrease in major diagnosis indicators by 0.23% points (95% CI –0.36% to –0.10%; *P<*.001). Conversely, the major treatment indicator experienced a significant increase of 0.57% points (95% CI 0.07%-1.07%; *P*=.03), and the upward transfer rate also significantly increased by 0.52% points (95% CI 0.02%-1.03%; *P*=.04). Moreover, there was a trend of increasing diagnostic fees, with a rise of 460.66 points (95% CI –3.44 to 924.76; *P*=.05). For STEMI cases, the diagnostic fees significantly increased by 2746.59 points (95% CI 1141.67-4351.51; *P<*.001). When examining septic shock, the major diagnosis indicator saw a significant decrease of 0.25% points (95% CI –0.37% to –0.12%; *P*<.001) following the introduction of the CHEC policy. Thirty-day mortality decreased by 0.11% (95% CI –0.20% to –0.02%; *P*=.02), and one-year mortality decreased by 0.15% (95% CI –0.22% to –0.07%; *P*<.001). In addition, medical orders significantly dropped by 9.67 items (95% CI –13.99 to –5.35; *P*<.001), and average medical expenses significantly decreased by 11,059.10 points (95% CI –18,603.60 to –3514.55; *P*=.004). Finally, regarding major trauma cases, after CHEC policy implementation, the average medical orders significantly decreased by 16.13 items (95% CI –25.32 to –6.94; *P*<.001).

**Table 4:** Association of categorization of hospital emergency capability (CHEC) policy with process and outcome quality in 4 critical time-sensitive diseases.

Critical time-sensitive disease		Change between before and after CHEC policy	Model 1^a^		Model 2^b^			
			*β*_2_ (95% CI)	*P* value	*β*_3_ (95% CI)		*P* value	
**Acute ischemic stroke^c^ (n=2895)**								
	Major diagnosis indicator, n (%)	–3.52	–0.23 (–0.36 to –0.10)	<.001		–0.06 (–0.32 to 0.20)		.66
	Diagnostic fees^d^	460.66	460.66 (–3.44 to 924.76)	.05		–302.19 (–1419.15 to 814.77)		.60
	Major treatment indicator, n (%)	0.62	0.57 (0.07 to 1.07)	*.03*		0.77 (0.21 to 1.33)		.007
	Medical orders per case	−0.93	–0.93 (–4.64 to 2.78)	.62		15.20 (5.28 to 25.11)		.003
	Upward transfer rate, n (%)	0.59	0.52 (0.02 to 1.03)	.04		0.21 (–0.39 to 0.81)		.49
	Short-term mortality (30 d), n (%)	–0.04	−0.01 (−0.25 to 0.23)	.95		0.11 (–0.27 to 0.49)		.57
	Long-term mortality (365 d), n (%)	0.28	0.02 (−0.12 to 0.16)	.77		0.06 (*–*0.22 to.033)		.69
	Total medical expense per case^h^	3616.16	3616.15 (*–*3524.26 to 10,756.56)	.32		16,672.69 (*–*3581.75 to 36,927.12)		.11
**ST-segment elevation myocardial infarction^e^(n=723)**								
	Major diagnosis indicator, n (%)	0.55	0.09 (*–*0.32 to 0.49)	.68		0.26 (*–*0.21 to 0.72)		.28
	Diagnostic fees	2746.6	2746.59 (1141.67 to 4351.51)	<.001		1983.75 (84.28 to 3883.21)		*.04*
	Major treatment indicator, n (%)	2.07	0.09 (–0.11 to 0.30)	.38		0.30 (–0.03 to 0.62)		.07
	Medical orders per case	−4.21	–4.21 (–13.14 to 4.72)	.36		11.92 (–0.90 to 24.73)		.07
	Upward transfer rate, n (%)	–1.24	–0.28 (–0.76 to 0.21)	.27		–0.59 (–1.18 to –0.001)		.049
	Short-term mortality (30 d), n (%)	–1.52	–0.10 (–0.36 to 0.16)	.45		–0.02 (–0.37 to 0.41)		.92
	Long-term mortality (365 d), n (%)	–0.83	−0.04 (−0.26 to 0.18)	.72		–0.01 (–0.33 to .032)		.97
	Total medical expense per case	11,219.01	11,219.00 (–4953.90 to 27,391.90)	.17		24,275.54 (–640.71 to 49,191.78)		.06
**Septic shock^f^ (n=5441)**								
	Major diagnosis indicator, n (%)	–2.28	−0.25 (−0.37 to −0.12)	<.001		–0.08 (–0.33 to 0.18)		.56
	Diagnostic fees	–44.8	–44.80 (–412.26 to 322.66)	.81		–807.65 (–1888.03 to 272.74)		.14
	Major treatment indicator, n (%)	–1.25	−0.06 (−0.14 to 0.02)	.14		0.14 (–0.12 to 0.41)		.28
	Medical orders per case	–9.67	–9.67 (–13.99 to –5.35)	<.001		6.45 (–3.70 to 16.61)		.21
	Upward transfer rate, n (%)	–0.1	–0.27 (–0.93 to 0.38)	.41		–0.59 (–1.32 to 0.15)		.12
	Short-term mortality (30 d), n (%)	–1.84	–0.11 (–0.20 to –0.02)	.02		–0.01 (–0.29 to 0.31)		.95
	Long-term mortality (365 d), n (%)	–3.64	–0.15 (–0.22 to –0.07)	<.001		–0.11 (–0.36 to 0.13)		.36
	Total medical expense per case	–11,059.09	–11,059.10 (–18,603.60 to –3514.55)	.004		1997.45 (–18,403.00 to 22,397.86)		.85
**Major trauma^g^ (n=864)**								
	Major diagnosis indicator, n (%)	–3.01	–0.17 (–0.39 to 0.05)	.14		N/A^h^		N/A
	Diagnostic fees	762.85	762.85 (–253.13 to 1778.82)	.14		N/A		N/A
	Major treatment indicator, n (%)	–3.12	–0.21 (–0.46 to 0.05)	.11		N/A		N/A
	Medical orders per case	–16.13	–16.13 (–25.32 to –6.94)	<.001		N/A		N/A
	Upward transfer rate, n (%)	2.43	0.31 (–0.02 to 0.65)	.06		N/A		N/A
	Short-term mortality (30 d), n (%)	–1.27	–0.12 (–0.41 to 0.17)	.43		N/A		N/A
	Long-term mortality (365 d), n (%)	–0.58	–0.03 (–0.27 to 0.20)	.77		N/A		N/A
	Total medical expense per case	−13,056.54	–13,056.50 (–32,010.60 to 5897.53)	.18		N/A		N/A

^a^Model 1 compares the specific disease differences between before and after CHEC policy implementation.

^b^Model 2: model-adjusted estimates for an interaction between a binary measure of CHEC policy (ie, postimplementation vs preimplementation) and critical time-sensitive diseases compared with major trauma (eg, acute ischemic stroke vs major trauma, ST-segment elevation myocardial infarction vs major trauma, and septic shock vs major trauma).

^c^Acute ischemic stroke major diagnosis indicator: head image and major treatment indicator: intravenous tissue plasminogen activator thrombolysis.

^d^Diagnostic fees: since Taiwan’s National Health Insurance system operates on a global budget with reimbursement based on a point system, the actual monetary value of each point fluctuates. Currently, 1 National Health Insurance point is reimbursed at <NT $0.90 (US $0.0275) per point based on the latest exchange rate (NT $1=US $0.03057 as of February 16, 2025).

^e^STEMI: ST-segment elevation myocardial infarction major diagnosis indicator: electrocardiography and major treatment indicator: percutaneous coronary intervention.

^f^Septic shock major diagnosis indicator: culture and major treatment indicator: antipathogen medication.

^g^Major trauma major diagnosis indicator: computed tomography, magnetic resonance imaging, or sonography study and major treatment indicator: rescue operation.

^h^N/A: data not applicable as major trauma cases were the reference group.

In model 2, results from the GEE model highlighted the CHEC policy’s varied effects across different diseases. A positive group-by-disease interaction β_3_ coefficient indicated that the outcome changes for that disease was greater than the reference group (Table 4). Compared to major trauma cases, AIS cases exhibited a significant increase in the major treatment indicator (interaction β coefficient=0.77; 95% CI 0.21-1.33; *P*=.007) and medical orders (interaction β coefficient=15.20; 95% CI 5.28-25.11; *P*=.003) between before and after CHEC policy implementation. Meanwhile, STEMI cases demonstrated a significant increase in diagnostic fees (interaction β coefficient=1983.75; 95% CI 84.28-3883.21; *P*=.04) and a significant decrease in upward transfer rate (interaction β coefficient=−0.59; 95% CI –1.18 to –0.001; *P*=.049) than the major trauma cases. Moreover, there were trends toward increasing major treatment indicators (interaction β coefficient=0.30; 95% CI –0.03 to 0.62; *P*=.07), medical orders (interaction β coefficient=11.92; 95% CI –0.90 to 24.73; *P*=.07), and medical expense (interaction β coefficient=24,275.54; 95% CI –640.71 to 4,991,991.78; *P*=.06), although these were not statistically significant. Compared to major trauma cases, no significant change was observed in either process or outcome quality indicators for septic shock cases.

## Discussion

### Principal Findings

The CHEC policy was universally implemented across Taiwan’s emergency medical service systems. Evaluating the impact of such a policy on a nationwide population presents significant challenges, primarily due to the absence of a control group. This limitation restricts the analysis to pre- and postimplementation comparisons, complicating the understanding of how the policy may influence shifts in various diseases. Despite these challenges, our study aimed to analyze the policy’s differential effects on time-sensitive conditions thoroughly. By examining disease-specific guidelines, we identified the groups most affected by the policy and those serving as relatively unaffected counterfactuals, offering valuable insights into the policy’s impact. To address these challenges, we used a DID design combined with PSM, using major trauma cases as a reference group. This approach ensured that baseline covariates were balanced between before and after policy periods, allowing us to investigate the policy’s effects while controlling for preexisting differences in participant and hospital characteristics. Our analysis revealed 2 major findings. First, the CHEC policy effectively improved system efficiency and patient outcomes, resulting in significant reductions in medical orders (ie, –7.29 items per case), short- and long-term mortality rates (ie, –0.09% each), and total medical expenses (ie, –5328.35 points per case), despite a modest increase in diagnostic fees (ie, 376.37 points). Second, we observed unintended “policy spotlight effects,” where conditions with time-based surveillance indicators, such as AIS and STEMI, showed disproportionately greater improvements than conditions without such indicators. Specifically, AIS cases experienced significant increases in major treatments (β=0.77) and medical orders (β=15.20), while STEMI cases demonstrated increased diagnostic fees (β=1983.75) and decreased upward transfer rates (β=–0.59) relative to major trauma cases. These findings highlight the varied impacts of the policy based on the presence or absence of time-based monitoring indicators. Thus, the effectiveness and efficiency of the CHEC policy underscore its dual ability to lower costs while improving patient outcomes. However, why has the CHEC policy significantly reduced medical costs and mortality rates, even though the major diagnosis indicator has declined, and no substantial changes have been observed in treatment indicators? The subsequent sections will delve further into diseases’ individual and interactive effects to provide analysis.

### Distinction Between the Hawthorne and Policy Spotlight Effects

To distinguish health care providers’ behaviors influenced by the Hawthorne effect or policy spotlight effects, we selected AIS and STEMI, which have well-established guidelines and time-based quality indicators under the CHEC policy. In contrast, septic shock, a disease with well-established guidelines but no specific time-based quality indicators, and major trauma, lacking both well-established guidelines and time-based quality indicators, were chosen as reference groups (Table S2 in Multimedia Appendix 1).

After implementing the CHEC policy, we hypothesized that the observed responses might indicate varying levels of awareness among emergency care providers [24]. The Hawthorne effect highlights that individuals’ productivity in experimental settings may increase due to the simple fact that they are being observed. This phenomenon underscores the influence of human attention and intervention on behavior [25]. The policy spotlight effects may be intensified by factors such as time constraints, ambiguous symptom patterns, and time-based quality surveillance indicators, prompting emergency care providers to unconsciously adopt selective behaviors concentrating on specific diseases according to policy targets [25]. Consequently, diseases not prioritized by the policy, such as septic shock and major trauma in this study, may experience a significant decline in process quality and a reduction in medical orders on an individual disease basis (Table 3). When using major trauma as the reference group, both AIS and STEMI group showed increases in major treatments, medical orders, diagnostic fees, and medical expenses. In contrast, no significant changes were observed in the septic shock group (Table 4).

When examining the effects of the policy on individual diseases, we observed distinct patterns. A decrease in diagnostic indicators was noted for AIS cases, likely due to increased upward transfer rates, as hospital physicians appeared more inclined to transfer patients with AIS to higher-level hospitals to enhance access to thrombolytic treatment [26]. This aligns with a significant increase in primary treatment indicators. For STEMI cases, there was an increase in diagnostic fees and a notable rise in diagnostic and treatment indicators, coupled with decreased transfer rates, suggesting a more intensive treatment approach that correlates with reduced mortality rates. These findings highlight the differential impacts of the policy, with AIS and STEMI cases benefiting from improvements in process and treatment quality due to the presence of time-based indicators and targeted monitoring. In contrast, septic shock and major trauma cases, which lack such indicators, showed either no significant changes or potential deterioration in quality metrics. These contrasting trends between diseases with and without time-based monitoring suggest the influence of policy spotlight effects. Under the CHEC policy, AIS and STEMI cases have demonstrated marked improvements, while septic shock and major trauma cases remain relatively unaffected, underscoring the importance of targeted policy monitoring in driving quality improvements.

While the Hawthorne effect typically manifests as generalized behavioral improvements across most observed conditions due to the awareness of being monitored, the implementation of the CHEC policy was explicitly designed to optimize emergency medical capacity through enhanced adherence to evidence-based guidelines, with a particular emphasis on reducing unnecessary medical orders and improving emergency care efficiency. The observed outcomes demonstrated significant improvements, including reductions in medical orders and per-case expenses, concurrent with decreased short- and long-term mortality rates, which are the overall impact of CHEC policy effects on the 4 CTSDs. These observed improvements may be attributed to the Hawthorne effect, wherein the awareness of being observed leads to enhanced performance among health care providers.

Both AIS and STEMI cases appeared to be influenced by the policy spotlight effect, and they showed opposite results in diagnostic indicators and upward transfer rates. These could potentially be attributed to the inherent differences between AIS and STEMI cases. For instance, only about 1% to 2% of all patients with AIS underwent primary treatment with intravenous thrombolytic agents, which is far lower than the STEMI major treatment rate. Unexpectedly, in less policy spotlight affected groups, such as septic shock and major trauma, despite significant decreases in primary diagnostic indicators, medical orders, and diagnostic fees, there were unexpected reductions in 30-day and 1-year mortality rates and medical expenses per event. These unintended consequences might resonate with the “less is more” initiative [27], suggesting that curbing excessive diagnoses and avoiding unnecessary procedures could improve patient outcomes and decrease costs [28,29].

### Policy Implications

Many emergency care policies implement time-based criteria [30-32], such as Australia’s 4-hour rule [30] and the United Kingdom’s 4-hour standard [32]. Australia’s experience showed that an emergency care policy using time-based criteria could improve emergency congestion without increasing the rate of ED revisits. However, in New Zealand, a policy in effect from 2006 to 2012 dictated that emergency patients must be hospitalized, transferred, or discharged within 6 hours of visiting the ED. After emergency care policy intervention, the length of ED stay decreased while the treatment outcomes of acute myocardial infarction, severe septic shock, and acute appendicitis did not improve significantly [31]. Similarly, after the Canadian Emergency Observation Reduction Program implementation, the length of ED stay decreased, while the treatment quality indicators for acute myocardial infarction, asthma, and upper limb fractures could only be treated in time for the abovementioned time-sensitive diseases during the noncongested emergency period [33].

Emergency care quality is closely related to the practices of medical care providers [12]. Policy makers must reconsider the conventional emphasis on time-based process indicators. This approach may have unintended consequences for diseases that are outside the “spotlight” of rigid time-sensitive evaluation. Instead, a broader and more nuanced evaluation of emergency care quality is needed to incorporate the complexity and ambiguity of various time-sensitive diseases that emergency care providers often manage [12]. We propose replacing time-based indicators with a broader set of multidimensional performance-based indicators, such as those exemplified in the National Health Service Best Practice Tariff policy [34]. This shift toward “best practice” can create a more flexible approach that goes beyond merely relying on time-based or diagnosis-based practices [34]. Such a transition is crucial, as an excessive reliance on diagnostic tests may lead to ED crowding, a decline in emergency care quality, and increased safety issues [28].

### Strengths

This study used a robust DID method within a GEE framework to evaluate the impact of the CHEC policy. The analysis compared pre- and postintervention periods and distinguished treatment and control groups using a categorical disease variable. Interaction terms captured differential policy effects across disease groups, while GEE accounted for correlated data from repeated measures, ensuring robust variance estimation. The estimated coefficients quantified both overall and disease-specific policy impacts, providing a reliable and nuanced evaluation of the CHEC policy. Our study also examined the behaviors of emergency care providers under time constraints and their interactions with strict time-based quality surveillance indicators and adherence to “get with the guidelines” protocols, revealing policy spotlight effects.

### Limitations

This study has several limitations. First, the data were derived from a secondary dataset of insurance claims rather than a randomized controlled trial, which may limit causal inferences. Second, the analysis lacked detailed information on time-related quality indicators, such as door-to-evaluation and door-to-treatment times, and critical emergency care metrics. Third, the effectiveness of emergency medical care often depends on collaboration between emergency and consulting physicians; however, this study did not explicitly evaluate the dynamics of their interaction. Future research should examine the coordination and teamwork between these physician groups to understand their impact on care quality better.

### Conclusions

Our study reveals that CHEC policy implementation demonstrates a dual capability to reduce costs and improve patient outcomes. The policy spotlight effects result in a disproportional improvement in disease guideline adherence and process quality of CTSDs with time-based surveillance indicators. In contrast, disease entities not fully encompassed in the surveillance indicators may be jeopardized by decreasing diagnosis and treatment process quality.

## Appendix

Multimedia Appendix 1 Propensity score–matched patient characteristics for 4 time-sensitive diseases before and after categorization of hospital emergency capability (CHEC) policy implementation, association of CHEC policy with process and outcome quality in 4 critical time-sensitive diseases, and the distinction between the Hawthorne and policy spotlight effects.

Multimedia Appendix 2 Association of CHEC policy with process and outcomes quality in four critical time-sensitive diseases.

## Abbreviations

AIS: acute ischemic stroke
CHEC: categorization of hospital emergency capability
CTSD: critical time-sensitive disease
DID: difference-in-differences
ED: emergency department
GEE: generalized estimating equation
ICD-9-CM: International Classification of Diseases, Ninth Revision, Clinical Modification
LHID2005: Longitudinal Health Insurance Database
PSM: propensity score matching
STEMI: ST-segment elevation myocardial infarction

## Data Availability

The data that support the findings of this study are available from the Taiwan National Health Insurance Research Database, but restrictions apply to the availability of these data, which were used under license for this study, and so are not publicly available. Data are, however, available at the academic request and with the permission of the Taiwan National Health Insurance Administration.
