# Supplementary material for: Policy Spotlight Effects on Critical Time-Sensitive Diseases: Nationwide Retrospective Cohort Study on Taiwan’s Hospital Emergency Capability Categorization Policy
Source: Interact J Med Res. 2025 Mar 25;14:e54651. doi: 10.2196/54651 (PMC11979550; doi:10.2196/54651)
Supplement: Multimedia Appendix 1 [file ijmr_v14i1e54651_app1.doc]

**Supplementary Table 1**. Propensity score matched patient characteristics for four time-sensitive diseases pre- and post-categorization of hospital emergency capability (CHEC) policy implementation

|  | **Acute ischemic stroke** | | | **ST-segment elevation MI^e^** | | | **Septic shock** | | | **Major trauma** | | |
| --- | --- | --- | --- | --- | --- | --- | --- | --- | --- | --- | --- | --- |
|  | Before CHEC policy (N=2895) | After CHEC policy (N=2895) | ASMD^a^ | Before CHEC policy (N=723) | After CHEC policy (N=723) | ASMD | Before CHEC policy (N=5441) | After CHEC policy (N=5441) | ASMD | Before CHEC policy (N=864) | After CHEC policy (N=864) | ASMD |
| **Sex** |  |  |  |  |  |  |  |  |  |  |  |  |
| Female | 1292 (44.63%) | 1220 (42.14%) | 0.050 | 221 (30.57%) | 213 (29.46%) | 0.024 | 2528 (46.46%) | 2511 (46.15%) | 0.006 | 316 (36.57%) | 324 (37.5%) | 0.019 |
| Male | 1603 (55.37%) | 1675 (57.86%) | 0.050 | 502 (69.43%) | 510 (70.54%) | 0.024 | 2913 (53.54%) | 2930 (53.85%) | 0.006 | 548 (63.43%) | 540 (62.5%) | 0.019 |
| **Age** |  |  |  |  |  |  |  |  |  |  |  |  |
| ≤ 45 | 129 (4.46%) | 123 (4.25%) | 0.010 | 64 (8.85%) | 61 (8.44%) | 0.015 | 822 (15.11%) | 845 (15.53%) | 0.012 | 274 (31.71%) | 277 (32.06%) | 0.008 |
| 45-64 | 798 (27.56%) | 815 (28.15%) | 0.013 | 242 (33.47%) | 247 (34.16%) | 0.015 | 1281 (23.54%) | 1306 (24%) | 0.011 | 229 (26.5%) | 222 (25.69%) | 0.018 |
| ≥ 65 | 1968 (67.98%) | 1957 (67.6%) | 0.008 | 417 (57.68%) | 415 (57.4%) | 0.006 | 3338 (61.35%) | 3290 (60.47%) | 0.018 | 361 (41.78%) | 365 (42.25%) | 0.010 |
| **Charlson Comorbidity Index** |  |  |  |  |  |  |  |  |  |  |  |  |
| ≤ 1 | 1594 (55.06%) | 1528 (52.78%) | 0.046 | 400 (55.33%) | 359 (49.65%) | 0.114 | 1865 (34.28%) | 1959 (36%) | 0.036 | 614 (71.06%) | 588 (68.06%) | 0.065 |
| ≥ 2 | 1301 (44.94%) | 1367 (47.22%) | 0.046 | 323 (44.67%) | 364 (50.35%) | 0.114 | 3576 (65.72%) | 3482 (64%) | 0.036 | 250 (28.94%) | 276 (31.94%) | 0.065 |
| **Income^b^** |  |  |  |  |  |  |  |  |  |  |  |  |
| <=22800 | 1326 (45.8%) | 1361 (47.01%) | 0.024 | 284 (39.28%) | 313 (43.29%) | 0.082 | 2406 (44.22%) | 2514 (46.2%) | 0.040 | 412 (47.69%) | 416 (48.15%) | 0.009 |
| >22800 | 1569 (54.2%) | 1534 (52.99%) | 0.024 | 439 (60.72%) | 410 (56.71%) | 0.082 | 3035 (55.78%) | 2927 (53.8%) | 0.040 | 452 (52.31%) | 448 (51.85%) | 0.009 |
| **Occupation** |  |  |  |  |  |  |  |  |  |  |  |  |
| Dependents of the insured persons | 1053 (36.37%) | 974 (33.64%) | 0.057 | 225 (31.12%) | 218 (30.15%) | 0.021 | 1984 (36.46%) | 1862 (34.22%) | 0.047 | 288 (33.33%) | 272 (31.48%) | 0.040 |
| Civil servants, teachers, military personnel | 302 (10.43%) | 322 (11.12%) | 0.022 | 81 (11.2%) | 80 (11.07%) | 0.004 | 533 (9.8%) | 591 (10.86%) | 0.035 | 70 (8.1%) | 60 (6.94%) | 0.044 |
| Nonmanual workers and professionals | 168 (5.8%) | 169 (5.84%) | 0.002 | 84 (11.62%) | 84 (11.62%) | 0.000 | 398 (7.31%) | 432 (7.94%) | 0.024 | 98 (11.34%) | 111 (12.85%) | 0.046 |
| Manual workers | 1042 (35.99%) | 1135 (39.21%) | 0.067 | 243 (33.61%) | 273 (37.76%) | 0.087 | 1853 (34.06%) | 1992 (36.61%) | 0.053 | 284 (32.87%) | 308 (35.65%) | 0.059 |
| Others | 330 (11.4%) | 295 (10.19%) | 0.039 | 90 (12.45%) | 68 (9.41%) | 0.098 | 673 (12.37%) | 564 (10.37%) | 0.063 | 124 (14.35%) | 113 (13.08%) | 0.037 |
| **Hospital categorization** |  |  |  |  |  |  |  |  |  |  |  |  |
| Severe level | 929 (32.09%) | 1034 (35.72%) | 0.077 | 237 (32.78%) | 266 (36.79%) | 0.084 | 1903 (34.98%) | 1908 (35.07%) | 0.002 | 262 (30.32%) | 294 (34.03%) | 0.079 |
| Moderate level | 1263 (43.63%) | 1205 (41.62%) | 0.041 | 281 (38.87%) | 269 (37.21%) | 0.034 | 2069 (38.03%) | 2183 (40.12%) | 0.043 | 417 (48.26%) | 395 (45.72%) | 0.051 |
| General level | 703 (24.28%) | 656 (22.66%) | 0.038 | 205 (28.35%) | 188 (26%) | 0.053 | 1469 (27%) | 1350 (24.81%) | 0.050 | 185 (21.41%) | 175 (20.25%) | 0.029 |
| **ESI^c^ triage level Ⅰ and Ⅱ rate** | 14.73 ± 10.87 | 15.08 ± 10.55 | 0.033 | 14.29 ± 11.64 | 14.65 ± 10.98 | 0.032 | 13.61 ± 10.03 | 14.02 ± 10.53 | 0.040 | 13.9 ± 9.71 | 13.97 ± 9.5 | 0.007 |
| **Length of ED^d^ stay** | 4.67 ± 6.66 | 4.23 ± 7.05 | 0.064 | 3.12 ± 5.31 | 2.85 ± 4.4 | 0.055 | 7.18 ± 10.36 | 6.05 ± 11.56 | 0.103 | 5.94 ± 8.25 | 4.35 ± 8.77 | 0.187 |
| **ED observation ≧ 1-day rate** | 10.4 ± 9.45 | 11.57 ± 10.31 | 0.118 | 10.83 ± 9.88 | 11.88 ± 10.78 | 0.102 | 10.7 ± 9.31 | 11.22 ± 10.04 | 0.054 | 9.64 ± 8.65 | 10.65 ± 9.35 | 0.112 |
| **Place of ED resources** |  |  |  |  |  |  |  |  |  |  |  |  |
| Sufficiency | 2245 (77.55%) | 2285 (78.93%) | 0.033 | 576 (79.67%) | 570 (78.84%) | 0.020 | 4264 (78.37%) | 4307 (79.16%) | 0.019 | 683 (79.05%) | 672 (77.78%) | 0.031 |
| Not sufficiency | 650 (22.45%) | 610 (21.07%) | 0.033 | 147 (20.33%) | 153 (21.16%) | 0.020 | 1177 (21.63%) | 1134 (20.84%) | 0.019 | 181 (20.95%) | 192 (22.22%) | 0.031 |
| **Care delivered by:** |  |  |  |  |  |  |  |  |  |  |  |  |
| Specialty Consultant^f^ | 1943 (67.12%) | 1860 (64.25%) | 0.060 | 411 (56.85%) | 400 (55.33%) | 0.031 | 3814 (70.1%) | 3788 (69.62%) | 0.010 | 570 (65.97%) | 522 (60.42%) | 0.115 |
| ED physician | 724 (25.01%) | 795 (27.46%) | 0.056 | 252 (34.85%) | 261 (36.1%) | 0.026 | 1226 (22.53%) | 1238 (22.75%) | 0.005 | 247 (28.59%) | 291 (33.68%) | 0.110 |
| Others | 228 (7.88%) | 240 (8.29%) | 0.015 | 60 (8.3%) | 62 (8.58%) | 0.010 | 401 (7.37%) | 415 (7.63%) | 0.010 | 47 (5.44%) | 51 (5.9%) | 0.020 |

^a^ASMD: absolute standardized mean difference.

^b^Income: 1 NT$ = 0.03057 US$ as of February 16, 2025.

^c^ESI: Emergency Severity Index.

^d^ED: emergency department.

^e^MI: myocardial infarction.

^f^Specialty consultant: (1) acute ischemic stroke is treated by neurologists, (2) acute MI is treated by cardiologists, (3) septic shock is managed by internal medicine physicians or critical care intensivists, and (4) major trauma conditions are managed by surgeons or critical care intensivists.

**TABLE 4** Association of CHEC policy with process and outcomes quality in four critical time-sensitive diseases

|  | **Before CHEC policy** | **After CHEC policy** | **Change between pre & post CHEC** | **Model 1^a^**  𝛽_2_ **(95% CI)^c^** | **P value** | **Model 2^b^**  𝛽_3_ **(95% CI)** | **P value** |
| --- | --- | --- | --- | --- | --- | --- | --- |
| **Acute ischemic stroke**^d^ **(N=2895)** |  |  |  |  |  |  |  |
| Major diagnosis indicator | 2391 (82.59%) | 2289 (79.07%) | **-3.52** | **-0.23 (-0.36 to -0.10)** | **0.0005** | -0.06 (-0.32 to 0.20) | 0.6635 |
| Diagnostic fees | 6050.63 (10934.56) | 6511.29 (6895.16) | **460.66** | **460.66 (-3.44 to 924.76)** | **0.0517** | -302.19 (-1419.15 to 814.77) | 0.5959 |
| Major treatment indicator | 24 (0.83%) | 42 (1.45%) | **0.62** | **0.57 (0.07 to 1.07)** | **0.0263** | **0.77 (0.21 to 1.33)** | **0.0068** |
| Medical orders per case | 70.44 (71.03) | 69.51 (74.08) | -0.93 | -0.93 (-4.64 to 2.78) | 0.6228 | **15.20 (5.28 to 25.11)** | **0.0027** |
| Upward transfer rate | 25 (0.86%) | 42 (1.45%) | **0.59** | **0.52 (0.02 to 1.03)** | **0.0399** | 0.21 (-0.39 to 0.81) | 0.4929 |
| Short-term mortality (30 days) | 140 (4.84%) | 139 (4.80%) | -0.04 | -0.01 (-0.25 to 0.23) | 0.951 | 0.11 (-0.27 to 0.49) | 0.5687 |
| Long-term mortality (365 days) | 441 (15.23%) | 449 (15.51%) | 0.28 | 0.02 (-0.12 to 0.16) | 0.7668 | 0.06 (-0.22 to .033) | 0.6874 |
| Total medical expense per case | 58995.28 (161260.94) | 62611.44 (113039.39) | 3616.16 | 3616.15 (-3524.26 to 10756.56) | 0.3209 | 16672.69 (-3581.75 to 36927.12) | 0.1067 |
| **ST-segment elevation MI**^e^ **(N=723)** |  |  |  |  |  |  |  |
| Major diagnosis indicator | 671 (92.81%) | 675 (93.36%) | 0.55 | 0.09 (-0.32 to 0.49) | 0.6767 | 0.26 (-0.21 to 0.72) | 0.2767 |
| Diagnostic fees | 6269.07 (11425.79) | 9015.67 (19940.85) | **2746.6** | **2746.59 (1141.67 to 4351.51)** | **0.0008** | **1983.75 (84.28 to 3883.21)** | **0.0407** |
| Major treatment indicator | 240 (33.20%) | 255 (35.27%) | 2.07 | 0.09 (-0.11 to 0.30) | 0.3824 | **0.30 (-0.03 to 0.62)** | **0.0729** |
| Medical orders per case | 92.31 (90.33) | 88.10 (88.05) | -4.21 | -4.21 (-13.14 to 4.72) | 0.3556 | **11.92 (-0.90 to 24.73)** | **0.068**4 |
| Upward transfer rate | 39 (5.39%) | 30 (4.15%) | -1.24 | -0.28 (-0.76 to 0.21) | 0.2654 | **-0.59 (-1.18 to -0.001)** | **0.0496** |
| Short-term mortality (30 days) | 144 (19.92%) | 133 (18.40%) | -1.52 | -0.10 (-0.36 to 0.16) | 0.4531 | -0.02 (-0.37 to 0.41) | 0.9247 |
| Long-term mortality (365 days) | 217 (30.01%) | 211 (29.18%) | -0.83 | -0.04 (-0.26 to 0.18) | 0.7227 | -0.01 (-0.33 to .032) | 0.9745 |
| Total medical expense per case | 108481.43 (144206.04) | 119700.44 (166898.18) | 11219.01 | 11219.00 (-4953.90 to 27391.90) | 0.174 | **24275.54 (-640.71 to 49191.78)** | **0.0562** |
| **Septic shock**^f^ **(N=5441)** |  |  |  |  |  |  |  |
| Major diagnosis indicator | 4941 (90.81%) | 4817 (88.53%) | **-2.28** | **-0.25 (-0.37 to -0.12)** | **<.0001** | -0.08 (-0.33 to 0.18) | 0.5573 |
| Diagnostic fees^h^ | 7360.02 (9079.06) | 7315.22 (10611.73) | -44.8 | -44.80 (-412.26 to 322.66) | 0.8111 | -807.65 (-1888.03 to 272.74) | 0.1429 |
| Major treatment indicator | 3894 (71.57%) | 3826 (70.32%) | -1.25 | -0.06 (-0.14 to 0.02) | 0.1434 | 0.14 (-0.12 to 0.41) | 0.2824 |
| Medical orders per case | 120.70 (124.08) | 111.03 (112.49) | **-9.67** | **-9.67 (-13.99 to -5.35)** | **<.0001** | 6.45 (-3.70 to 16.61) | 0.2129 |
| Upward transfer rate | 21 (0.39%) | 16 (0.29%) | -0.1 | -0.27 (-0.93 to 0.38) | 0.4125 | -0.59 (-1.32 to 0.15) | 0.1165 |
| Short-term mortality (30 days) | 1234 (22.68%) | 1134 (20.84%) | **-1.84** | **-0.11 (-0.20 to -0.02)** | **0.0189** | -0.01 (-0.29 to 0.31) | 0.9539 |
| Long-term mortality (365 days) | 2398 (44.07%) | 2200 (40.43%) | **-3.64** | **-0.15 (-0.22 to -0.07)** | **0.0001** | -0.11 (-0.36 to 0.13) | 0.3588 |
| Total medical expense per case | 115832.23 (206098.13) | 104773.14 (198516.37) | **-11059.09** | **-11059.10 (-18603.60 to -3514.55)** | **0.0041** | 1997.45 (-18403.00 to 22397.86) | 0.8478 |
| **Major trauma**^g^ **(N=864)** |  |  |  |  |  |  |  |
| Major diagnosis indicator | 679 (78.59%) | 653 (75.58%) | -3.01 | -0.17 (-0.39 to 0.05) | 0.1359 | Major trauma as reference group^i^ |  |
| Diagnostic fees | 10442.74 (10411.94) | 11205.59 (11322.87) | 762.85 | 762.85 (-253.13 to 1778.82) | 0.1411 |  |  |
| Major treatment indicator | 176 (20.37%) | 149 (17.25%) | -3.12 | -0.21 (-0.46 to 0.05) | 0.1086 |  |  |
| Medical orders per case | 99.56 (103.19) | 83.43 (93.90) | **-16.13** | **-16.13 (-25.32 to -6.94)** | **0.0006** |  |  |
| Upward transfer rate | 63 (7.29%) | 84 (9.72%) | **2.43** | **0.31 (-0.02 to 0.65)** | **0.0650** |  |  |
| Short-term mortality (30 days) | 113 (13.08%) | 102 (11.81%) | -1.27 | -0.12 (-0.41 to 0.17) | 0.4286 |  |  |
| Long-term mortality (365 days) | 186 (21.53%) | 181 (20.95%) | -0.58 | -0.03 (-0.27 to 0.20) | 0.7710 |  |  |
| Total medical expense per case | 140654.57 (215834.78) | 127598.03 (194545.71) | -13056.54 | -13056.50 (-32010.60 to 5897.53) | 0.1770 |  |  |
|  |  |  |  |  |  |  |  |

^a^Model 1: compares the specific disease differences between before and after CHEC policy implementation

^b^Model 2: the model adjusted estimates for an interaction between a binary measure of CHEC policy (ie, postimplementation vs preimplementation) and critical time-sensitive diseases compared with major trauma (eg, acute ischemic stroke vs major trauma, ST-segment elevation myocardial infarction vs major trauma, and septic shock vs major trauma)

^c^CI: confidence interval

^d^Acute ischemic stroke major diagnosis indicator: head image and major treatment indicator: intravenous tissue plasminogen activator thrombolysis.

^e^STEMI: ST-segment elevation myocardial infarction major diagnosis indicator: electrocardiography and major treatment indicator: percutaneous coronary intervention.

^f^Septic shock major diagnosis indicator: culture and major treatment indicator: antipathogen medication.

^g^Major trauma major diagnosis indicator: computed tomography, magnetic resonance imaging, or sonography study and major treatment indicator: rescue operation.

^h^Diagnostic fees: Since Taiwan’s National Health Insurance (NHI) system operates on a global budget with reimbursement based on a point system, the actual monetary value of each point fluctuates. Currently, one NHI point is reimbursed at less than NT$0.9 (US$0.0275) per point, based on the latest exchange rate (1 NT$ = 0.03057 US$ as of February 16, 2025).

^i^N/A: data not applicable as major trauma cases were the reference group.

Supplemental Table 3 The distinction between the Hawthorne and policy spotlight effects

| Criteria | Hawthorne Effect | Policy Spotlight Effects |
| --- | --- | --- |
| Focus | General observational improvements | Disease-specific, policy-targeted |
| Driver | Awareness of observation | Policy monitoring and time-based indicators |
| Impacts | Assessing post- and pre-policy effects on overall four CTSDs cohorts | Specific to AIS and STEMI (group-by-disease interaction effects) |
| Reference | Pre-policy period | Major trauma |
| Outcomes | Process and treatment quality  Outcomes | Process and treatment quality  Outcomes |
| Evidence^a,b^ | Decrease medical orders  Decrease mortality  Decrease medical expenses | Increase AIS major treatment  Increase AIS medical orders  Increase STEMI diagnostic fees  Increase STEMI major treatment  Increase STEMI medical orders  Decrease STEMI transfer rate  Increase STEMI medical expenses  Septic shock group no significant change |

^a^Hawthorne Effect evidence from Table 3: Comparative analysis of the overall impact of CHEC policy effects on four critical time-sensitive diseases (𝛽_1_)

^b^Policy Spotlight Effects evidence from Table 4: Association of CHEC policy with process and outcomes quality in four critical time-sensitive diseases (𝛽_3_)

AIS: acute ischemic stroke; CTSDs: critical time-sensitive diseases; STEMI: ST-segment elevation myocardial infarction
