# Supplementary material for: Policy Spotlight Effects on Critical Time-Sensitive Diseases: Nationwide Retrospective Cohort Study on Taiwan’s Hospital Emergency Capability Categorization Policy
Source: Interact J Med Res. 2025 Mar 25;14:e54651. doi: 10.2196/54651 (PMC11979550; doi:10.2196/54651)
Supplement: Multimedia Appendix 2 [file ijmr_v14i1e54651_app2.docx]

**Supplementary Table 2:** Association of CHEC policy with process and outcomes quality in four critical time-sensitive diseases

|  | **Before CHEC policy** | **After CHEC policy** | **Change between pre & post CHEC** | **Model 1^a^**  𝛽_2_ **(95% CI)^c^** | **P value** | **Model 2^b^**  𝛽_3_ **(95% CI)** | **P value** |
| --- | --- | --- | --- | --- | --- | --- | --- |
| **Acute ischemic stroke**^d^ **(N=2895)** |  |  |  |  |  |  |  |
| Major diagnosis indicator | 2391 (82.59%) | 2289 (79.07%) | **-3.52** | **-0.23 (-0.36 to -0.10)** | **0.0005** | -0.06 (-0.32 to 0.20) | 0.6635 |
| Diagnostic fees | 6050.63 (10934.56) | 6511.29 (6895.16) | **460.66** | **460.66 (-3.44 to 924.76)** | **0.0517** | -302.19 (-1419.15 to 814.77) | 0.5959 |
| Major treatment indicator | 24 (0.83%) | 42 (1.45%) | **0.62** | **0.57 (0.07 to 1.07)** | **0.0263** | **0.77 (0.21 to 1.33)** | **0.0068** |
| Medical orders per case | 70.44 (71.03) | 69.51 (74.08) | -0.93 | -0.93 (-4.64 to 2.78) | 0.6228 | **15.20 (5.28 to 25.11)** | **0.0027** |
| Upward transfer rate | 25 (0.86%) | 42 (1.45%) | **0.59** | **0.52 (0.02 to 1.03)** | **0.0399** | 0.21 (-0.39 to 0.81) | 0.4929 |
| Short-term mortality (30 days) | 140 (4.84%) | 139 (4.80%) | -0.04 | -0.01 (-0.25 to 0.23) | 0.951 | 0.11 (-0.27 to 0.49) | 0.5687 |
| Long-term mortality (365 days) | 441 (15.23%) | 449 (15.51%) | 0.28 | 0.02 (-0.12 to 0.16) | 0.7668 | 0.06 (-0.22 to .033) | 0.6874 |
| Total medical expense per case | 58995.28 (161260.94) | 62611.44 (113039.39) | 3616.16 | 3616.15 (-3524.26 to 10756.56) | 0.3209 | 16672.69 (-3581.75 to 36927.12) | 0.1067 |
| **ST-segment elevation MI**^e^ **(N=723)** |  |  |  |  |  |  |  |
| Major diagnosis indicator | 671 (92.81%) | 675 (93.36%) | 0.55 | 0.09 (-0.32 to 0.49) | 0.6767 | 0.26 (-0.21 to 0.72) | 0.2767 |
| Diagnostic fees | 6269.07 (11425.79) | 9015.67 (19940.85) | **2746.6** | **2746.59 (1141.67 to 4351.51)** | **0.0008** | **1983.75 (84.28 to 3883.21)** | **0.0407** |
| Major treatment indicator | 240 (33.20%) | 255 (35.27%) | 2.07 | 0.09 (-0.11 to 0.30) | 0.3824 | **0.30 (-0.03 to 0.62)** | **0.0729** |
| Medical orders per case | 92.31 (90.33) | 88.10 (88.05) | -4.21 | -4.21 (-13.14 to 4.72) | 0.3556 | **11.92 (-0.90 to 24.73)** | **0.068**4 |
| Upward transfer rate | 39 (5.39%) | 30 (4.15%) | -1.24 | -0.28 (-0.76 to 0.21) | 0.2654 | **-0.59 (-1.18 to -0.001)** | **0.0496** |
| Short-term mortality (30 days) | 144 (19.92%) | 133 (18.40%) | -1.52 | -0.10 (-0.36 to 0.16) | 0.4531 | -0.02 (-0.37 to 0.41) | 0.9247 |
| Long-term mortality (365 days) | 217 (30.01%) | 211 (29.18%) | -0.83 | -0.04 (-0.26 to 0.18) | 0.7227 | -0.01 (-0.33 to .032) | 0.9745 |
| Total medical expense per case | 108481.43 (144206.04) | 119700.44 (166898.18) | 11219.01 | 11219.00 (-4953.90 to 27391.90) | 0.174 | **24275.54 (-640.71 to 49191.78)** | **0.0562** |
| **Septic shock**^f^ **(N=5441)** |  |  |  |  |  |  |  |
| Major diagnosis indicator | 4941 (90.81%) | 4817 (88.53%) | **-2.28** | **-0.25 (-0.37 to -0.12)** | **<.0001** | -0.08 (-0.33 to 0.18) | 0.5573 |
| Diagnostic fees^h^ | 7360.02 (9079.06) | 7315.22 (10611.73) | -44.8 | -44.80 (-412.26 to 322.66) | 0.8111 | -807.65 (-1888.03 to 272.74) | 0.1429 |
| Major treatment indicator | 3894 (71.57%) | 3826 (70.32%) | -1.25 | -0.06 (-0.14 to 0.02) | 0.1434 | 0.14 (-0.12 to 0.41) | 0.2824 |
| Medical orders per case | 120.70 (124.08) | 111.03 (112.49) | **-9.67** | **-9.67 (-13.99 to -5.35)** | **<.0001** | 6.45 (-3.70 to 16.61) | 0.2129 |
| Upward transfer rate | 21 (0.39%) | 16 (0.29%) | -0.1 | -0.27 (-0.93 to 0.38) | 0.4125 | -0.59 (-1.32 to 0.15) | 0.1165 |
| Short-term mortality (30 days) | 1234 (22.68%) | 1134 (20.84%) | **-1.84** | **-0.11 (-0.20 to -0.02)** | **0.0189** | -0.01 (-0.29 to 0.31) | 0.9539 |
| Long-term mortality (365 days) | 2398 (44.07%) | 2200 (40.43%) | **-3.64** | **-0.15 (-0.22 to -0.07)** | **0.0001** | -0.11 (-0.36 to 0.13) | 0.3588 |
| Total medical expense per case | 115832.23 (206098.13) | 104773.14 (198516.37) | **-11059.09** | **-11059.10 (-18603.60 to -3514.55)** | **0.0041** | 1997.45 (-18403.00 to 22397.86) | 0.8478 |
| **Major trauma**^g^ **(N=864)** |  |  |  |  |  |  |  |
| Major diagnosis indicator | 679 (78.59%) | 653 (75.58%) | -3.01 | -0.17 (-0.39 to 0.05) | 0.1359 | Major trauma as reference group^i^ |  |
| Diagnostic fees | 10442.74 (10411.94) | 11205.59 (11322.87) | 762.85 | 762.85 (-253.13 to 1778.82) | 0.1411 |  |  |
| Major treatment indicator | 176 (20.37%) | 149 (17.25%) | -3.12 | -0.21 (-0.46 to 0.05) | 0.1086 |  |  |
| Medical orders per case | 99.56 (103.19) | 83.43 (93.90) | **-16.13** | **-16.13 (-25.32 to -6.94)** | **0.0006** |  |  |
| Upward transfer rate | 63 (7.29%) | 84 (9.72%) | **2.43** | **0.31 (-0.02 to 0.65)** | **0.0650** |  |  |
| Short-term mortality (30 days) | 113 (13.08%) | 102 (11.81%) | -1.27 | -0.12 (-0.41 to 0.17) | 0.4286 |  |  |
| Long-term mortality (365 days) | 186 (21.53%) | 181 (20.95%) | -0.58 | -0.03 (-0.27 to 0.20) | 0.7710 |  |  |
| Total medical expense per case | 140654.57 (215834.78) | 127598.03 (194545.71) | -13056.54 | -13056.50 (-32010.60 to 5897.53) | 0.1770 |  |  |
|  |  |  |  |  |  |  |  |

^a^Model 1: compares the specific disease differences between before and after CHEC policy implementation

^b^Model 2: the model adjusted estimates for an interaction between a binary measure of CHEC policy (ie, postimplementation vs preimplementation) and critical time-sensitive diseases compared with major trauma (eg, acute ischemic stroke vs major trauma, ST-segment elevation myocardial infarction vs major trauma, and septic shock vs major trauma)

^c^CI: confidence interval

^d^Acute ischemic stroke major diagnosis indicator: head image and major treatment indicator: intravenous tissue plasminogen activator thrombolysis.

^e^STEMI: ST-segment elevation myocardial infarction major diagnosis indicator: electrocardiography and major treatment indicator: percutaneous coronary intervention.

^f^Septic shock major diagnosis indicator: culture and major treatment indicator: antipathogen medication.

^g^Major trauma major diagnosis indicator: computed tomography, magnetic resonance imaging, or sonography study and major treatment indicator: rescue operation.

^h^Diagnostic fees: Since Taiwan’s National Health Insurance (NHI) system operates on a global budget with reimbursement based on a point system, the actual monetary value of each point fluctuates. Currently, one NHI point is reimbursed at less than NT$0.9 (US$0.0275) per point, based on the latest exchange rate (1 NT$ = 0.03057 US$ as of February 16, 2025).

^i^N/A: data not applicable as major trauma cases were the reference group.
